# Processibility, Thermo-Mechanical Properties, and Radiation Hardness of Polyurethane and Silicone Resins

**DOI:** 10.3390/polym17162240

**Published:** 2025-08-18

**Authors:** Christian Scheuerlein, Melanie Albeck, Roland Piccin, Federico Ravotti, Giuseppe Pezzullo

**Affiliations:** European Organization for Nuclear Research (CERN), Esplanade des Particules 1, 1211 Geneva, Switzerland

**Keywords:** Polyurethane, silicone, casting, elastomer, irradiation, superconducting magnet, cross-linking, chain scission

## Abstract

Different polyurethanes (PURs) and silicone for potential use in particle accelerators and detectors have been characterized in the uncured state, after curing, and after exposure to ionizing irradiation in ambient air and in liquid helium. The viscosity evolution during processing was measured with a rheometer. Dynamic mechanical analysis (DMA) and Shore A hardness measurements were applied to detect irradiation-induced crosslinking and chain scission effects. Uniaxial tensile and flexural tests under ambient and cryogenic conditions have been performed to assess changes in mechanical strength, elongation at break, and elastic properties. The initial viscosity of 550 cP at 25 °C of the uncured PUR RE700-4 polyol and RE106 isocyanate system for protective encapsulation is sufficiently low for impregnation of small magnet coils, but the pot life of about 30 min is too short for impregnation of large magnet coils. The cured RE700-4 system has outstanding mechanical properties at 77 K (flexural strength, impact strength, and fracture toughness). When RE700-4 is exposed to ionizing radiation, chain scission and cross-linking occur at a similar rate. In the other casting systems, irradiation-induced changes are cross-linking dominated, as manifested by an increase of the rubbery shear modulus (*G’_rubbery_*), the ambient temperature Young’s modulus (*E_RT_*), and the Shore A hardness. Cross-linking rates are strongly reduced when irradiation occurs in liquid helium. The irradiation effect on mechanical properties can be strongly dependent on the testing temperature. The RT mechanical strength and strain at fracture of the cross-linking silicone is drastically decreased after 1.6 MGy, whereas its 77 K strain at fracture has almost doubled. In addition, 77 K elastic moduli are similar for all pure resins and only slightly affected by irradiation.

## 1. Introduction

To qualify polymers for possible applications in particle accelerators and detectors, the processability of the uncured resin mix, the thermomechanical properties of the cured resins, and their resistance to ionizing irradiation [1,2,3] need to be characterized. Irradiation can occur under different environments, ranging from ambient air to in a vacuum or in inert gas and at very low temperatures.

Polyurethanes (PURs) can be used for instance for potting and encapsulation of electronics devices and as adhesive. PUR elastomers are used as load bearing material in accelerator magnet alignment jacks. Room temperature vulcanizing (RTV) silicones can be used for sealing applications, and special-low temperature RTV grades retain their elastomeric flexibility also at cryogenic temperatures.

In the present study, three PUR systems for protective encapsulation of electrical circuits and electronic devices (Sika RE700-4, Sika RE820 and Robnor ResinLab EL110H) and two PUR elastomers that exhibit high elongation and high mechanical strength (Sika UR350, and Pad ShA75) have been characterized. The latter have been studied as possible materials in magnet alignment jacks in the CERN accelerator complex [4,5]. For comparison, the silicone elastomer Silastic RTV-4250-S that is used for fabrication of special seals and molds has been added to this study.

Superconducting magnet coils made of Nb_3_Sn superconductors will be used in the magnets of future accelerators like those of the High Luminosity Large Hadron Collider (HL-LHC) [6,7,8], the Future Circular Collider (FCC) [9], or the Muon Collider [10]. These coils need to be impregnated with a resin [11].

PUR systems contain high-molecular-weight constituents that make the viscosity of the uncured resin rather high. In addition, the pot life of typical PURs is relatively short. Therefore, PUR systems are usually not considered for impregnation of large superconducting magnet coils.

However, the outstanding low temperature mechanical properties of some PURs [12] make them interesting candidate materials for superconducting coil impregnation, provided that the processibility of the uncured resin allows the material to fill the entire void space between the conductors of the coil and that the cured resin properties are maintained when exposed to ionizing radiation.

Viscosity has been measured as a function of temperature as well as duration at constant temperature. Dynamical mechanical analysis (DMA) has been performed to measure the temperature dependent viscoelastic properties of the cured samples. Irradiation-induced aging has been assessed using Shore A hardness measurements, tensile tests at room temperature (RT), flexural tests at 77 K, and impact tests at 77 K, both before irradiation and after irradiation in ambient air. Since the irradiation environment and the irradiation temperature influence the efficiency of the radiation source to produce radiation damage [13], 10 MGy irradiation for one polyurethane sample was also performed in liquid helium. It is shown that irradiation-induced cross-linking is largely reduced when irradiation occurs at cryogenic temperature.

## 2. Materials and Methods

### 2.1. The Samples

Here, 4 mm thick plates of six different PUR systems and one silicone plate have been produced at the CERN polymer laboratory using vacuum impregnation. The PUR plate referred to as “Pad 75 ShA” has been received in cast form.

Samples for tensile stress–strain, fracture toughness, flexural strength, and dynamic mechanical analysis (DMA) tests were cut out from the plates with water jet cutting. All samples of a given material were cut from the same plate, thus eliminating uncertainties related to the sample production processes.

#### 2.1.1. SikaBiresin^®^ RE 700-4

The two component SikaBiresin^®^ RE 700-4 polyol (100 pbw)/SikaBiresin^®^ (Sika AG, Baar, Switzerland) RE 106 isocyanate (100 pbw) system is a RT curing soft transparent polyurethane used for electronic component protection. The RE700-04 mixture has a comparatively low viscosity of 200 cP at 25 °C, which makes it a candidate material for magnet coil impregnation [14]. At 77 K, it has a very high fracture toughness of *K*_Q_ = 7 MPa√m [12].

#### 2.1.2. SikaBiresin^®^ RE 820

The black casting resin SikaBiresin^®^ RE 820 polyether polyol (100 pbw) and RE102 isocyanate (25 pbw) is a RT curing flexible polyurethane system used for protective dielectric encapsulation of electronic devices [15].

#### 2.1.3. Robnor ResinLab EL110H

Robnor ResinLab EL110H (Swindon, UK) is a black, RT curing, two-part flexible polyurethane, consisting of the RL110H resin (100 pbw) and the HL110H hardener (27 pbw). It is recommended for potting electronic components, especially for high-frequency, high-voltage applications [16].

#### 2.1.4. SikaBiresin^®^ UR350

The black casting elastomer SikaBiresin^®^ UR350 (previously known as UR3450) [17] consists of the colorless isocyanate UR350 part A (100 pbw) and the black polyester aliphatic polyol UR350 part B (35 pbw), which have been cured for 12 h at 40 °C. UR350 has been considered for pads in magnet alignment jacks [4].

#### 2.1.5. Pad ShA75

Pad ShA75 is a cast polyether-based polyurethane elastomer. The Shore A hardness of 75 can be adjusted by varying the concentrations of the three components. Of note, Ø = 50 mm ShA75 pads are used in magnet alignment jacks [4] to carry loads up to 6 tons.

#### 2.1.6. Silastic RTV-4250-S

The room temperature vulcanizing (RTV) Silastic RTV-4250-S (green) is a cross-linking two-part silicone rubber, which main application is the fabrication of flexible molds.

### 2.2. Viscosity Measurements

Viscosity measurements were performed with a Lamy Rheology RM-100-CP2000 Plus cone–plate viscometer (Champagne-au-Mont-d’Or, France) operated in speed control, using a Ø = 40 mm plate (CP4005). The shear rate was 120 s^−1^. For data acquisition, the RheoTex VX software was used. Viscosity was measured as a function of duration at constant temperature or as a function of temperature during a temperature ramp rate of 9 °C/min.

### 2.3. Irradiation

Exposure to ionizing irradiation was realized with a gamma ray source and a high-energy proton beam. Provided that the dose rate, irradiation environment, and irradiation temperature are similar, the effect of both sources can be compared using the absorbed dose as a scaling factor [18].

#### 2.3.1. Gamma Irradiation

Gamma irradiation with a ^60^Co source was performed at the Gammatec facility at the Marcoule site of the company Synergy Health Marseille SAS, France, with a dose rate of about 2 kGy/h in ambient air at a temperature of 20–25 °C. During irradiation, the sample holders were continuously rotated for better dose homogeneity. More details about the irradiation procedures can be found in [18].

#### 2.3.2. Proton Irradiation

Proton irradiation has been achieved at the CERN IRRAD facility with a pulsed 24 GeV/c proton beam from the CERN PS accelerator, delivering about 7 × 10^11^ protons per pulse of ~400 ms length. The average proton fluence of about 1.4 × 10^16^ p/cm^2^ per week corresponds to a dose of about 4 MGy per week in samples of 10 mm × 10 mm cross-section. Proton irradiation has been performed in ambient air at a temperature of 21 °C in inert gas at ambient temperature under a continuous nitrogen flow of 200 L/h as well as in liquid helium, using a dedicated cryostat installed in IRRAD. More details about the proton irradiation runs can be found in [13].

### 2.4. Dynamic Mechanical Analysis (DMA)

Storage modulus (*G*′) and loss modulus (*G*″) were recorded during temperature sweeps with a MCR702e dynamical mechanical analyzer from Anton Paar, Graz, Austria. Glass transition temperatures have been derived according to ASTM D 4065, DIN EN ISO 11357 [19,20], at a frequency of 1 Hz and a temperature ramp of 2 K/min. Irradiation-induced changes of the rubbery shear modulus (*G′_rubbery_*) have been determined as a measure of the molecular weight between the cross-links and the cross-link density [21,22,23].

### 2.5. Tensile Stress–Strain Measurements

Uniaxial tensile stress–strain measurements based on ISO 527 [24] have been performed using DIN 50125-E 3 × 8 × 30 samples, with a constant crosshead speed of 100 mm/min. Strain is measured with an extensometer with a gauge length of 10 mm. The engineering stress is calculated from the cross-section of the unloaded samples.

### 2.6. Flexural Tests

Three-point bending flexural tests were conducted according to ISO 178 [25], with rectangular beams of 80 mm × 10 mm and a nominal thickness of 4 mm, using Ø = 10 mm loading supports and bending die. The span length was 16× the nominal sample thickness, and the crosshead speed was 2 mm/min. The flexural modulus is determined as the slope of the linear fit in the strain range 0.05 to 0.25%. The flexural stress and flexural strain are calculated according to Equations (1) and (2) from the load (*F*), the specimen width (*b*), the specimen thickness (*h*), the support span (*L*), and the displacement (*s*) as follows:

(1) $$Flexural\;stress\;(\mathrm{MPa}):\;\sigma =\;\frac{3\times F\times L}{\begin{array}{c} 2\times b\times h^{2} \end{array}}$$

(2) $$Flexural\;strain\;(\%):\;\varepsilon =\frac{6\times s\times h}{L^{2}}\times 100\;\%$$

### 2.7. Impact Tests

Impact tests at 77 K were performed in Dynstat configuration according to DIN 53435 [26], which enables tests with comparatively small samples. In the present study, unnotched samples with nominal thickness of 4 mm, a width of 10 mm, and 20 mm length have been used. Tests were conducted on a Zwick and Roell HIT5.5P (Zwick GmbH & Co. KG, Ulm, Germany) impact tester with a 2 J pendulum. Before the impact test, the samples were cooled in liquid nitrogen at 77 K.

The impact strength (*a_dD_*) in kJ/m^2^ has been calculated from the energy absorbed for breaking the sample (*E_c_*), the sample thickness (*h*), and the sample width (*b*) according to (3):

(3) $$a_{dD}=\frac{E_{c}}{h\times b}\times 10^{3}$$

### 2.8. Shore A Hardness

Irradiation dose-dependent Shore A hardness values were derived using a Zwick durometer. The penetration of the indenter into the 4 mm thick specimen is measured under a load of 1 kg after a dwell time of 3 s. The results presented are the arithmetic mean of five measurements.

## 3. Results

### 3.1. Viscosity of the Uncured Silicone and Polyurethanes

**Figure 1:**
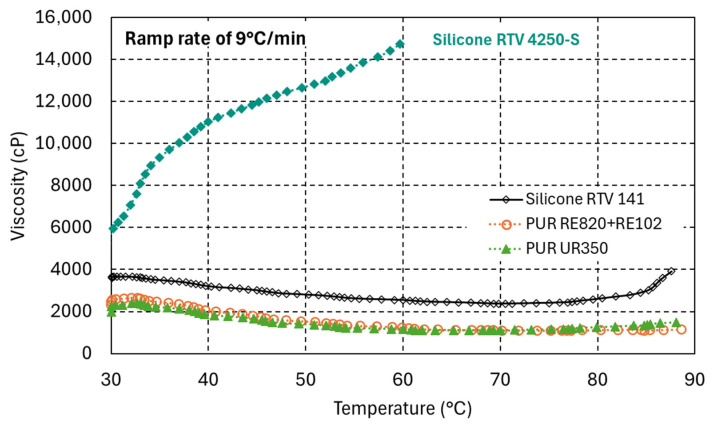
Viscosity evolution as a function of temperature > 30 °C during a temperature ramp of 9 °C/min.

The viscosity temperature dependence of the silicone RTV 4250-S and the PUR RE820 and UR350 has been measured during a 9 °C/min temperature ramp (Figure 1). The viscosity of the PUR decreases with increasing temperature due to increased molecular mobility and reduced intermolecular forces. In contrast, the silicone viscosity immediately increases with increasing temperature, presumably because thermal cross-linking is dominant already at comparatively lower temperature.

**Figure 2:**
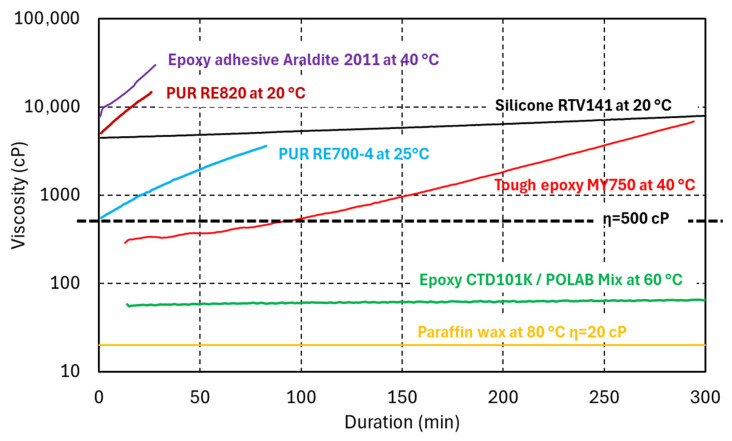
Comparison of the viscosity evolution of thermosetting polyurethanes and epoxy resins as a function of duration and viscosity of paraffin wax at their processing temperatures. The viscosity measurements of RE 700-4, the epoxy resins, and wax are courtesy of C. Urscheler.

In Figure 2, the viscosity evolutions of the PUR RE820 and RE700-4 systems are compared with those of epoxy resin systems considered for coil impregnation as well as epoxy adhesive, a low-viscosity silicone, and wax. It is assumed that during superconducting coil impregnation, the viscosity of the resin should not rise much above η = 500 cP (dashed line in Figure 2).

The RE700-4 viscosity of 550 cP at 25 °C is sufficiently low for impregnation of small magnet coils. However, the pot life of about 30 min is relatively short as compared to that of the epoxy resin systems used for coil impregnation. Alternative PUR systems with lower viscosity are commercially available and will be studied.

### 3.2. Viscoelastic Properties of the Cured Resins

The viscoelastic properties of the cured resins have been investigated using DMA in torsion mode. The effect of ionizing irradiation on cross-linking and chain scission has been derived from storage modulus *G’*(T) and loss modulus *G’’*(T) evolutions. The glass transition is manifested by a drop of *G*’ of about 2 orders of magnitude. The rubbery shear modulus (*G’_rubber_*) is measured when the glass transition temperature has been exceeded and is a measure for the cross-link density.

**Figure 3:**
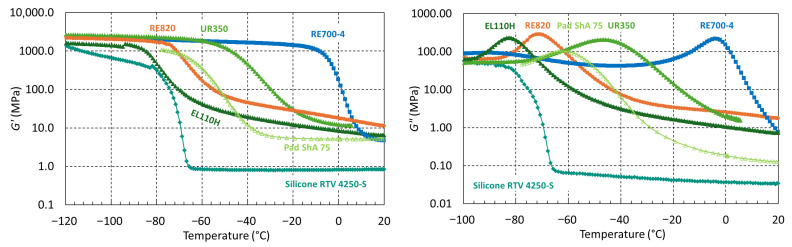
Storage modulus (*G*’) and loss modulus (*G*’’) of silicone and polyurethanes as a function of temperature.

In Figure 3, the storage and loss moduli of the non-irradiated elastomers are plotted as a function of temperature. All elastomers tested have their glass transition below RT and are in the rubbery state at ambient temperature. The Sika RE700-4 system has the highest glass transition temperature (*T_g_ G’’_max_* = −5 °C). Silicone RTV 4250-S has a lower rubbery modulus (*G’_rubbery_* < 1 MPa), indicating that it is less cross-linked than the PUR systems, in which *G’_rubber_* is in the order of 10 MPa.

### 3.3. Irradiation-Induced Changes in Shore A Hardness

**Figure 4:**
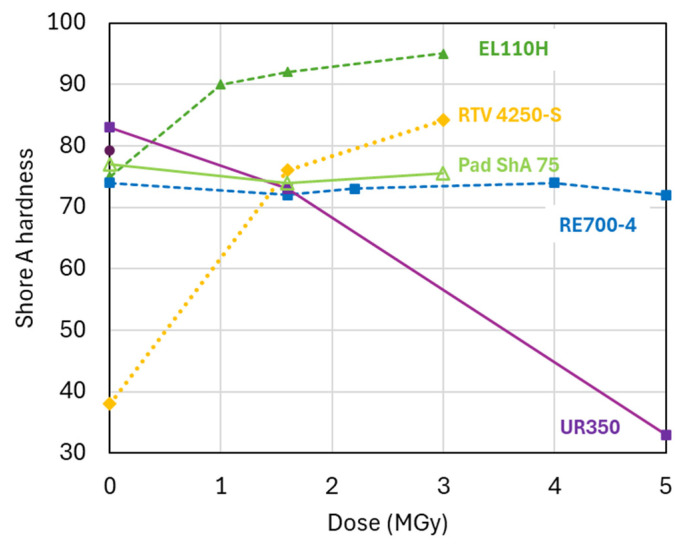
Shore A hardness as a function of the dose absorbed in ambient air. PUR for casting, PUR elastomers, and silicone elastomers are represented by dashed lines, continues lines, and dotted lines, respectively.

In Figure 4, the Shore A hardness evolution of the PUR and silicone systems is plotted as a function of the gamma dose absorbed in ambient air. Irradiation-induced cross-linking causes an increase of the two silicone systems. Similarly, irradiation-induced cross-linking is observed for the EL110H and RE820 systems, in which hardness, *G’_rubbery_*, and *E_RT_* increase with increasing dose.

The initial hardness of the RE700-4 system of 74 ± 1 Shore A 3 s remains nearly constant up to a dose of 5 MGy, suggesting similar irradiation-induced cross-linking and chain scission rates. Further increasing the dose to 10 MGy increases the hardness to 87 ± 0.6 Shore A 3 s.

The PUR system UR350 is chain scission dominated, as seen by the decrease of hardness, *G’_rubbery_*, and of *E_RT_*. After a dose of 1.6 MGy, the initial EL110H hardness of 75 ± 0.5 Shore A 3 s is increased to 92 ± 3 Shore A 3 s, and the hardness continues to increase with increasing dose.

Table 1 compares *T_g_*, *G’_rubbery_*, and the Shore A hardness measured before and after 1.6 MGy irradiation. In an ideal rubber, the cross-link density is proportional to *G’_rubbery_*. *G’_rubbery_* and Shore A results show that for the silicone, PUR RE820, and PUR EL110H, irradiation effects after 1.6 MGy are cross-linking dominated, whereas they are chain scission dominated for the PUR UR350. The cross-link density of RE700-4 and Pad ShA75 is not strongly changed after 1.6 MGy, suggesting that cross-linking and chain scission occur at similar rates.

### 3.4. Irradiation-Induced Changes in Mechanical Properties

The effect of 1.6 MGy irradiation on mechanical properties was assessed using tensile tests performed at RT (above *T_g_*), flexural tests with the samples immersed in liquid nitrogen at 77 K (below *T_g_*), and impact tests at 77 K. Before irradiation, all materials resist RT to 77 K temperature cycles by immersion in liquid nitrogen. After 1.6 MGy irradiation, however, the PUR UR350 disintegrates when immersed in LN_2_, and mechanical properties at 77 K could not be determined. For the PUR Pad ShA 75, no irradiated samples suitable for tensile testing were available.

**Figure 5:**
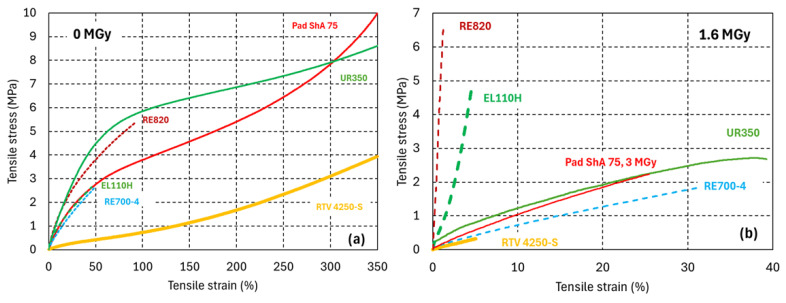
Uniaxial tensile engineering stress–strain curves of silicone and PURs (**a**) before and (**b**) after 1.6 MGy gamma irradiation. PURs for casting are represented by dashed lines, and the PUR and silicone elastomers are represented by continuous lines. The PUR elastomer Pad ShA 75 has been tested after a dose of 3.0 MGy.

The RT tensile stress–strain curves of the PUR and silicone before and after irradiation to 1.6 MGy are presented in Figure 5a,b, respectively. Before irradiation, the PUR for casting applications (EL110H, RE700-4, and RE820) break at comparatively low tensile strain. In contrast, the PUR elastomers and the silicon RTV 4250-S break at very high tensile strain, exceeding 350%.

After a gamma dose of 1.6 MGy absorbed in ambient air, the mechanical strength of the elastomers is drastically degraded, mainly due to breakage of covalent bonds in the polymer backbone.

The PUR for protective casting exhibit either strong irradiation-induced cross-linking and become rigid at RT (RE820 and EL110H) or *E_RT_* remains nearly unchanged (RE700-4 *E_RT_* = 7 MPa).

After exposure to 1.6 MGy, the RT tensile strain at fracture (*ɛ_max-RT_*) of RE700-4 is halved to about 30%. Thus, according to [27], the RE700-4 dose limit would be 1.6 MGy. The same dose drastically reduces the *ɛ_max-RT_* of RE820 and EL110H from 95% to <1% and from 45% to 5%, respectively.

**Figure 6:**
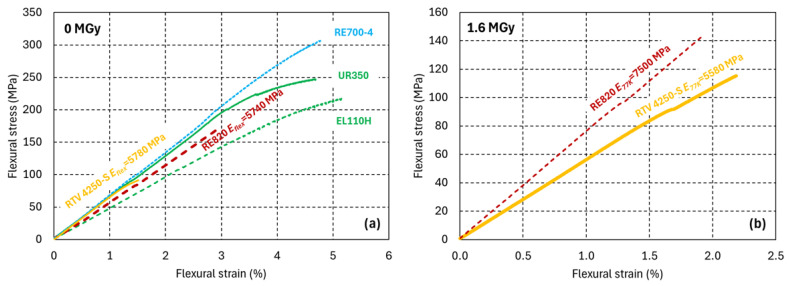
77 K flexural stress–strain curves of silicone and PUR (**a**) before and (**b**) after 1.6 MGy gamma irradiation. PURs for casting are represented by dashed lines, and the PUR and the silicone elastomers are represented by continues lines.

The 77 K flexural stress–strain results are shown in Figure 6. At 77 K, the silicone and PUR have similar elastic moduli between 4.5 GPa and 7.5 GPa. Irradiation-induced cross-linking and chain scission have a comparatively small influence on the 77 K elastic properties, which are determined by the van der Waals forces between the macromolecules [12,28]. The PUR RE700-4 has the highest 77 K flexural strength.

**Figure 7:**
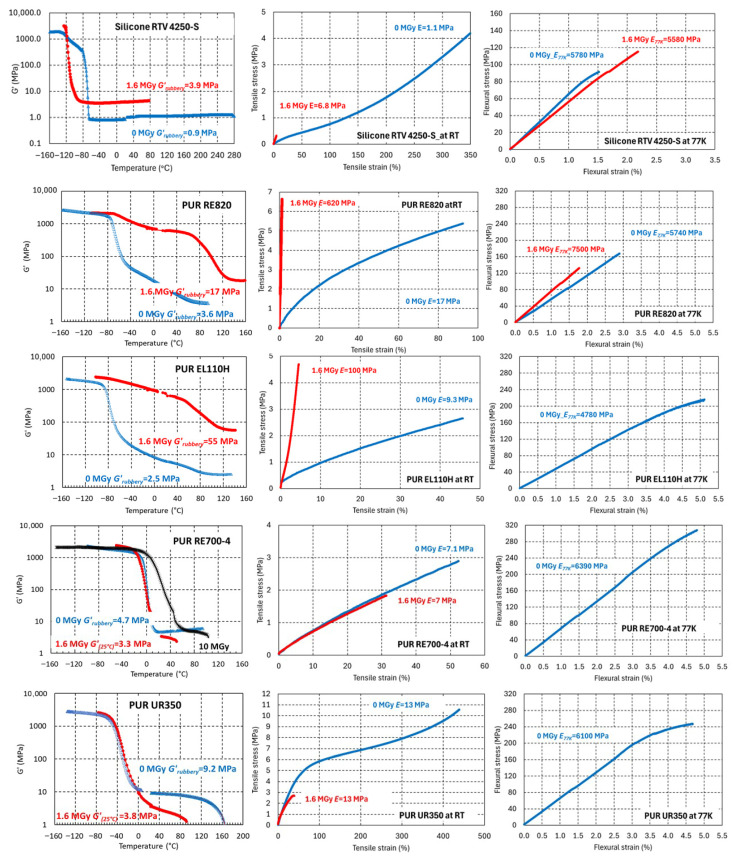
*G*’(T), RT uniaxial tensile engineering stress–strain and 77 K flexural stress–strain curves of silicone elastomer RTV 4250-S, the PUR systems for protective encapsulation, RE820, EL110H, RE700-4, and the UR350 elastomer before and after 1.6 MGy gamma irradiation.

Figure 7 compares *G*’(T), tensile properties at RT, and the flexural properties at 77 K before and after 1.6 MGy gamma irradiation of the Silicone RTV 4250-S, the three PUR systems for protective encapsulation (RE820, EL110H, and RE-700-4), and the PUR elastomer UR350.

The irradiation effect on mechanical properties of the cross-linking silicone RTV 4250-S is strongly dependent on the test temperature. After exposure to 1.6 MGy absorbed in ambient air, its *ɛ_max-RT_* is drastically reduced by 96%, whereas *ɛ_max-77K_* is increased by 48% with respect to those of the non-irradiated silicone.

Table 2 summarizes the main differences observed before and after 1.6 MGy irradiations. Clear changes in *G’_rubbery_*, *E_RT_*, and Shore A indicate that in silicone and the PUR RE820 and EL110H irradiation effects are cross-linking dominated. In the PUR elastomer UR350, they are clearly chain-scission dominated. In PUR RE700-4 and Pad ShA75, changes in *G’_rubbery_*, *E_RT_*, and Shore A are comparatively small, suggesting that up to 1.6 MGy in these materials cross-linking and chain scission occur at similar rates.

### 3.5. Irradiation-Induced Changes of 77 K Impact Strength

**Figure 8:**
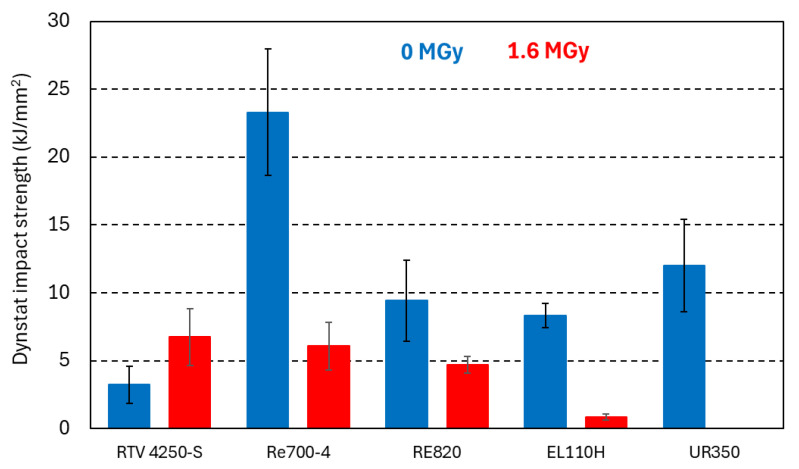
Unnotched Dynstat impact strength of PUR and silicone at 77 K before (blue bars) and after 1.6 MGy irradiation (red bars).

In Figure 8, the 77 K impact strengths of the different PUR and silicone samples are compared before and after 1.6 MGy irradiation. Impact tests were performed in Dynstat configuration with unnotched samples, immediately after removing the samples from liquid nitrogen.

Before irradiation, the RE700-4 system has outstandingly high impact strength of 23 kJ/mm^2^. After a dose of 2.2 MGy absorbed in ambient air, the RE700-4 impact strength at 77 K is reduced to 6.1 kJ/mm^2^. Further increasing the dose to 10 MGy reduces the RE700-4 impact strength to 3.3 kJ/mm^2^.

The impact strength of silicone RTV 4250-S is increased after 1.6 MGy irradiation.

After 1.6 MGy, UR350 samples disintegrate when immersed in liquid nitrogen, and their impact strength could not be determined.

### 3.6. Effect of Irradiation Atmosphere and Temperature on Irradiation-Induced Cross-Linking

**Figure 9:**
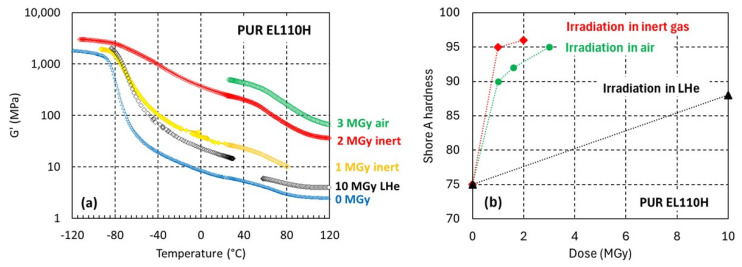
PUR EL110H (**a**) *G’*(T) and (**b**) Shore A hardness before irradiation and after proton irradiation in different environments (10 MGy in LHe, 1 MGy and 2 MGy in inert gas, and 3 MGy in ambient air).

In addition to the ambient air irradiation, the cross-linking PUR EL110H has also been irradiated in liquid helium (LHe) at 4 K and in inert gas at 21 °C. The effect of the irradiation temperature can be assessed from the *G’*(T) evolutions shown in Figure 9a.

A dose of 10 MGy absorbed in LHe increases the rubbery modulus of EL110H by 60% from ***G’_rubbery-0 MGy_*** = 2.5 MPa to ***G’_rubbery-10 MGy-LHe_*** = 4.0 MPa. After a dose of only 3 MGy absorbed in ambient air, the cross-link density is 15 times higher (***G’_rubbery-3MGy-air_ ***is increased to 60 MPa).

The strong effect of the irradiation temperature is confirmed by the dose-dependent Shore A hardness results presented in Figure 9b. The EL110H hardness increase to 88 ± 0.6 Shore A 3 s after 10 MGy absorbed in liquid helium is smaller than the hardness measured after 1 MGy absorbed in inert gas at ambient temperature (90 Shore A 3 s), confirming that at least for the EL110H system cross-linking is drastically reduced when irradiation occurs at very low temperatures.

## 4. Discussion and Conclusions

The PUR systems of this study have been investigated for two main purposes: (1) protective encapsulation of electronic components and electric devices like small superconducting magnet coils, and (2) mechanical support of accelerator magnets in harsh ambient air environment where the PUR is loaded up to compressive stresses of 50 MPa.

PUR are polymers containing urethane –NHCOO– groups in the polymeric chain resulting from the reaction between polyols and diisocyanates, and their properties are determined by the type of the polyols and the isocyanates from which they are composed. Most of the technical data sheets do not state the polyol and isocyanate types. However, the characteristics of the different PUR can suggest what polyol type they are based on. For electronic encapsulation, polyether-based PUR are typically used because they exhibit superior hydrolytic stability as compared to PUR with polyester polyol [29]. Polyether-based PUR can also be available with comparatively lower viscosity of the uncured resin, which is required for applications where the resin needs to easily flow and penetrate small porosities.

Processibility is a main challenge for the application of PURs in magnet coils. Among the materials tested in this study, only the RE700-4 system viscosity is initially sufficiently low, but its pot life of 30 min limits the possible application RE700-4 to small coils, for which impregnation or casting duration is relatively short. The impregnation of large coils like those of the HL-LHC magnets can last several hours [30], and resins with lower viscosity and longer pot life are needed. When a larger quantity of PUR is mixed, a possible temperature increase of the liquid resin due to the exothermic curing process might further reduce the duration during which the resin can be processed. Alternative low viscosity PUR systems for protective encapsulation and casting are commercially available and will be tested.

Other impregnation resin requirements for superconducting magnet coils include their dielectric and mechanical properties from ambient temperature down to temperatures as low as 1.9K. The hardness and rigidity of elastomers increases with decreasing temperature, and they become brittle below their glass transition temperature. The *T_g_* onset of the PURs of this study is in the range −90 °C (EL110H) to −10 °C (RE700-4). At RT the PUR elastomers have by definition much higher mechanical strength and strain at fracture than the casting resins.

At 77 K, however, the mechanical properties of the casting resins are comparable, or even superior to those of the PUR elastomers. The RE700-4 system has the highest 77 K flexural strength and impact strength of the materials of this study. RE700-4 exhibits also outstandingly high fracture toughness at 77 K [12], exceeding the fracture toughness of all epoxy resin systems typically used for superconducting magnet coil impregnation.

For the application of PUR systems in particle accelerators and detectors, the effect of ionizing irradiation needs to be verified. Polyether-based PURs typically exhibit lower chain scission rates and are more resistant to radiation [31]. The two PUR RE820 and EL110H casting resins (presumably polyether based) are irradiation-induced cross-linking dominated, as manifested by a strong increase in *E_RT_* as well as increases in the Shore A hardness and *G’_rubbery_*.

The initially nearly constant *E_RT_*, *G’_rubbery_*, and Shore A hardness values of the RE700-4 system suggest that cross-linking and chain scission occur at a similar rate in this resin. Assuming that the dose limit is the dose at which the strain at fracture is halved [27], the RE700-4 dose limit is about 1.6 MGy. For the polyester-based UR350 elastomer, irradiation-induced chain scission causes a reduction in *G’_rubbery_* and *E_RT_*.

A possible drawback of polyether-based PURs can be their oxidative degradation, especially at elevated temperatures. This needs to be considered when they are used in oxygen-containing environments.

When applied in superconducting magnets, polymers are exposed to ionizing irradiation at temperatures down to 1.9 K in the absence of oxygen. At such low temperatures, the movement of molecules and free radicals is drastically reduced, which makes it much less likely that radicals on different chains can react and form new bonds between chains.

As an example, a dose of 10 MGy absorbed in liquid He has less effect on the PUR EL110H materials properties of *G’_rubbery_* and ShA hardness than a dose of 1 MGy absorbed in inert gas. Thus, the low temperature dose limit of the EL110H system is >10 times higher than the ambient temperature dose limit.

Since the irradiation temperature affects the mobility of free radicals and the irradiation-induced cross-linking and chain scission rates, irradiation under cold conditions is mandatory to precisely determine the dose limits of polymers in superconducting magnets.

## Figures and Tables

**Table 1:** Glass transition temperature (*T_g_*), rubbery modulus (*G’_rubbery_*), and Shore A hardness of PURs and silicone before and after 1.6 MGy irradiation in ambient air. Not measured (n.m.).

Material	Dose (MGy)	*T_g_* (°C)			*G’_rubbery_* at 25 °C (MPa)	Shore A Hardness
		*G’*_onset_	*G’’*_max_	tan δ_max_		
Silicone RTV 4250-S	0	n.m.	−122	−118	0.8	38 ± 0.4
	1.6	−119	−120	−114	3.9	76 ± 2
PUR RE700-4	0	−5.1	−4.2	5.8	4.7	74 ± 1
	1.6	n.m.	−12	5.1	3.3	72 ± 1
PUR RE820	0	−75	−72	−63	3.6 (at 75 °C)	80 ± 0.4
	1.6	n.m.	−53	−40	17 (at 140 °C)	>100
PUR EL110H	0	−92	−83	−76	2.5	75 ± 0.5
	1.6	n.m.	−66	−12	55 (at 125 °C)	92 ± 4
PUR UR350	0	−51	−47	−27	9.2	83 ± 1
	1.6	n.m.	−46	−15	3.5	73 ± 1
PUR Pad ShA 75	0	n.m.	−64	−46	6.0	78 ± 0.5
	1.6	n.m.	−65	−43	4.9	71 ± 4

**Table 2:** Comparison of materials properties before and after a dose of 1.6 MGy in ambient air ^[b]^ Tacky after 1.6 MGy. ^[c]^ Very strong RE820 hardness increase after 1.6 MGy, exceeding the Shore A scale. ^[d]^ UR350 samples disintegrate when immersed in liquid nitrogen. Not measured (n.m.).

Material	*G’_rubbery_*	*E_RT_*	*Shore A*	*ɛ_max_ at RT*	*ɛ_max_ at 77 K*
Silicone RTV 4250-S cross-linking dominated	+490%	+520%	+95%	−96%	+48%
RE700-4 ^[b]^	−30%	<5%	<5%	−45%	n.m.
RE820 cross-linking dominated	+350%	+3500%	n.m. ^[c]^	−99%	−38%
EL110H cross-linking dominated	+690%	+1000%	+23%	−90%	n.m.
UR350 ^[b]^ (polyester-based polyol) chain-scission dominated	−62%	+6%	−12%	−90%	n.m. ^[d]^
Pad ShA 75 (polyether-based polyol)	−18%	<5%	<5%	n.m.	n.m.

## Data Availability

The original contributions presented in this study are included in the article. Further inquiries can be directed to the corresponding author.
